# Function and Assembly of a Chromatin-Associated RNase P that Is Required for Efficient Transcription by RNA Polymerase I

**DOI:** 10.1371/journal.pone.0004072

**Published:** 2008-12-30

**Authors:** Robert Reiner, Natalie Krasnov-Yoeli, Yana Dehtiar, Nayef Jarrous

**Affiliations:** Department of Molecular Biology, The Hebrew University-Hadassah Medical School, Jerusalem, Israel; Institute of Genetics and Molecular and Cellular Biology, France

## Abstract

**Background:**

Human RNase P has been initially described as a tRNA processing enzyme, consisting of H1 RNA and at least ten distinct protein subunits. Recent findings, however, indicate that this catalytic ribonucleoprotein is also required for transcription of small noncoding RNA genes by RNA polymerase III (Pol III). Notably, subunits of human RNase P are localized in the nucleolus, thus raising the possibility that this ribonucleoprotein complex is implicated in transcription of rRNA genes by Pol I.

**Methodology/Principal Findings:**

By using biochemical and reverse genetic means we show here that human RNase P is required for efficient transcription of rDNA by Pol I. Thus, inactivation of RNase P by targeting its protein subunits for destruction by RNA interference or its H1 RNA moiety for specific cleavage causes marked reduction in transcription of rDNA by Pol I. However, RNase P restores Pol I transcription in a defined reconstitution system. Nuclear run on assays reveal that inactivation of RNase P reduces the level of nascent transcription by Pol I, and more considerably that of Pol III. Moreover, RNase P copurifies and associates with components of Pol I and its transcription factors and binds to chromatin of the promoter and coding region of rDNA. Strikingly, RNase P detaches from transcriptionally inactive rDNA in mitosis and reassociates with it at G1 phase through a dynamic and stepwise assembly process that is correlated with renewal of transcription.

**Conclusions/Significance:**

Our findings reveal that RNase P activates transcription of rDNA by Pol I through a novel assembly process and that this catalytic ribonucleoprotein determines the transcription output of Pol I and Pol III, two functionally coordinated transcription machineries.

## Introduction

Transcription is carried out by functionally distinct nuclear RNA polymerases (pols) associated with general transcription factors, as well as specificity and coregulatory factors that assist in formation and function of preinitiation complexes. Pol I transcribes rRNA genes, Pol II mainly synthesizes protein-coding genes, while Pol III transcribes a large set of small noncoding RNA genes. Recent findings reveal that noncoding RNAs associate with and regulate pols I, II and III [1]–[6]. Thus, U1 snRNA and 7SK RNA regulate initiation and elongation of transcription by Pol II [7], [8], Alu RNA represses transcription by binding to Pol II in response to heat shock [9], IGS RNA facilitates silencing of Pol I transcription of rRNA genes through interaction with the chromatin remodeling complex NoRC [3], while the H1 RNA subunit of human nuclear RNase P is required for Pol III transcription of small noncoding RNA genes [10], [11]. These noncoding RNAs act in the context of ribonucleoprotein complexes [3], [10], [12].

Human nuclear RNase P has been initially characterized as a tRNA processing ribonucleoprotein, consisting of H1 RNA and at least ten distinct protein subunits, termed Rpp14, Rpp20, Rpp21, Rpp25, Rpp29, Rpp30, Rpp38, Rpp40, hPop1 and hPop5 [13]. The endonucleolytic activity of human RNase P in tRNA processing requires its H1 RNA entity, which recognizes precursor tRNA as substrate [14]. A recent work reports that H1 RNA mediates cleavage of precursor tRNA in the absence of protein [15]. Accordingly, the main input of the numerous protein subunits of human RNase P should be in other complex and versatile tasks of this ribonucleoprotein complex, e. g. transcription [11], [16], as will be further corroborated in this study.

We have previously demonstrated that human nuclear RNase P is required for transcription of small noncoding RNA genes transcribed by Pol III [10], [11]. RNase P exerts its role in transcription through association with Pol III and with chromatin of Pol III genes, including the 5S rRNA genes whose transcripts are not known to be processed by RNase P [10]. RNase P acts as an auxiliary factor for Pol III, as is the case with the transcription factors TFIIIA, TFIIIB and TFIIIC. This latter concept is based on the fact that Pol III can catalyze transcription reactions in a simplified in vitro transcription system in the absence of TFIIIB and TFIIIC that facilitate reinitation of transcription [17]. Moreover, Pol III requires only TFIIIB for transcription of tRNA and 5S rRNA genes in vitro [18], [19] and a highly purified human Pol III combined with recombinant SNAPc and TFIIIB subunits can direct multiple cycles of in vitro transcription initiation and termination from a U6 snRNA gene promoter [20].

H1 RNA is an abundant molecule in the cell. This transcript was detected in the cytoplasm, nucleoplasm and nucleoli. Protein subunits of human RNase P have also been differentially detected in specialized intranuclear compartments associated with active gene transcription, including nucleoli [16]. Mass spectrometry analysis of highly purified nucleoli of human cells confirms the existence of many protein subunits of RNase P, including Rpp14, Rpp20, Rpp25, Rpp29, Rpp30, Rpp38, Rpp40 and hPop1, in these bodies [21]. Indirect immunofluorescent analyses demarcate some of these protein subunits in confined sub-nucleolar sites, such as Rpp29 that resides in the dense fibrillar component, in which transcription and processing of rRNA take place [22], [23]. RNase P shares its protein subunits with the nucleolar ribonucleoprotein RNase MRP, except for the subunits Rpp21 and H1 RNA, which could be used to discriminate between the two ribonucleoproteins [24]. The exact role of human RNase MRP in the nucleolus remains unknown while its yeast counterpart is required for processing of 5.8S rRNA [25].

In this study, we show by biochemical and reverse genetic means that H1 RNA and its protein subunits, as part of an RNase P ribonucleoprotein, are required for efficient transcription of rDNA by Pol I. RNase P copurifies and associates with components of Pol I and its transcription initiation factors and exerts its role in transcription through association with the promoter and coding region of rDNA. Furthermore, we demonstrate that RNase P disengages from rDNA in mitosis and reassociates with it at G1 phase through a dynamic and stepwise assembly process. Our data implicate a catalytic ribonucleoprotein in transcription by Pol I and Pol III, whose coordinated function is critical for protein synthesis and cell growth.

## Results

### Knockdown of protein subunits of human RNase P inhibits Pol I function

**Figure 1:**
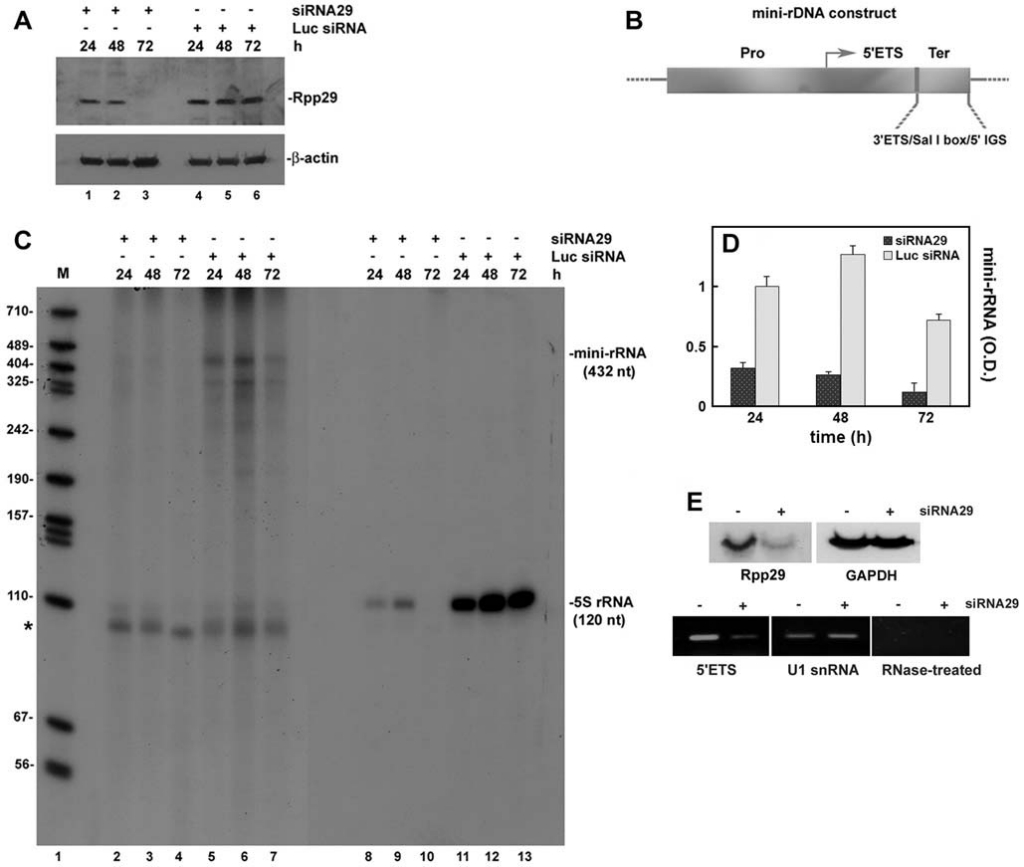
Knockdown of Rpp29 by siRNA causes inhibition of Pol I transcription. A. HeLa cells were transfected with siRNA29 or luciferase siRNA (see Materials and Methods), whole extracts were then prepared from cells at 24, 48 and 72 h after transfection and examined by Western blot analysis for Rpp29 and β-actin. Positions of proteins are depicted. B. A scheme of the human mini-rDNA gene. This construct has a rDNA gene promoter (from position −500) and 5′ETS (position +382) that was subcloned in pBluescript in which a rDNA terminator region, consisting of 57-bp 3′ETS, Sal I box and 51-bp IGS, was inserted. The 5′ETS is 382 bp in length and harbors no known cleavage site, including the A′ site. Arrow points to the transcription initiation site. C. Transcription of the mini-rRNA and 5S rRNA genes in extracts described in A. Labeled RNA was separated in 8% polyacrylamide/7 M urea gel and subjected to autoradiography. Lanes 1–7 represent exposure for longer time than that of lanes 8–13. Positions of the 432-nt mini-rRNA (lanes 2–7) and 120-nt 5S rRNA (lanes 8–13) transcripts are shown. Asterisk represents endogenous but unknown labeled RNA. D. The optical density (in arbitrary units) of the mini-rRNA band seen in lanes 2–7 of panel C. E. HeLa cells were treated with siRNA29 or luciferase siRNA for 48 h and knockdown of Rpp29 was tested by Western blot analysis (left panel). GAPDH was used as internal control. Total RNA was extracted from cells and subjected to RT-PCR analysis of 5′ETS and U1 snRNA (see Materials and Methods). As negative control, RNA was treated with RNase A before reverse transcription of the 5′ETS (right panel). PCR products were separated in agarose gels.

Rpp29 was targeted for destruction in HeLa cells by the use of RNA interference. Western blot analysis revealed efficient knockdown of Rpp29 in cells transfected with siRNA29 [26] but not with luciferase siRNA (Figure 1A, lanes 1–3 versus 4–6). Transcription of a human mini-rDNA gene, which is composed of rDNA promoter and adjacent 5′ETS segment fused to a terminator region (Figure 1B), took place in the control whole cell extracts (Figure 1C, lanes 5–7) but not in those prepared from cells treated with siRNA29 (Figure 1C, lanes 2–4; Figure 1D). Similarly, transcription of a human 5S rRNA gene was deficient in the extracts derived from cells with knockdown of Rpp29 (Figure 1C, lanes 8–10 versus 11–13), as previously shown [10]. Moreover, knockdown of Rpp29 by siRNA29 (Figure 1E) caused a marked decrease in 45S pre-rRNA synthesis in cells, as determined by RT-PCR (Figure 1E). The primers used for detection of the pre-rRNA recognize the 5′ETS, which is processed quickly from the primary transcript during transcription and thus reflects transcription initiation by Pol I. By contrast, expression of the U1 snRNA, which is transcribed by Pol II, remained unchanged (Figure 1E).

**Figure 2:**
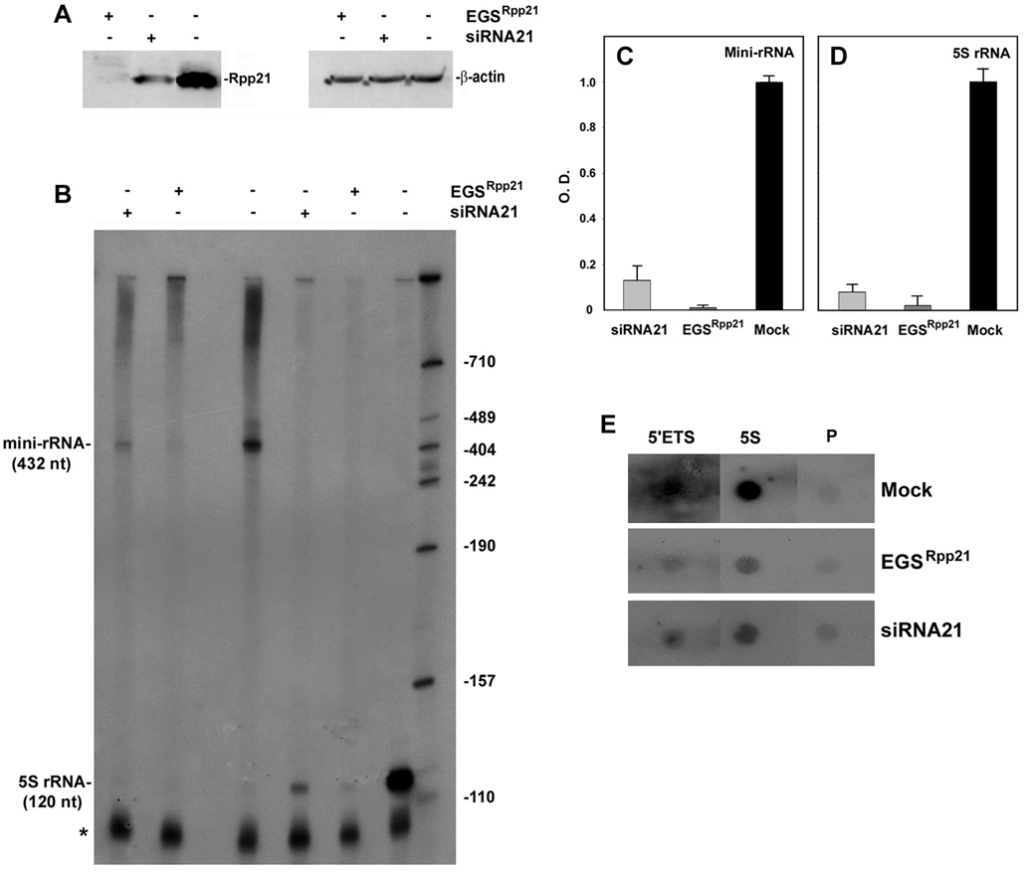
Knockdown of the specific subunit Rpp21 inhibits transcription and pre-rRNA synthesis. A. HeLa cells were transfected with a plasmid expressing EGS^Rpp21^ or siRNA21 or mocked transfected for 48 h. Whole HeLa extracts were then prepared and tested for the presence of Rpp21 and β-actin by Western blot analysis. B. Transcription of the mini-rRNA and 5S rRNA genes in extracts described in A and the reaction products were analyzed as in Figure 1C. C and D. The bands of the mini-rRNA and 5S rRNA were quantitated and plotted. E. Nuclear run-on transcription analysis (see Material and Methods) of the human 5′ETS rRNA and 5S rRNA genes in mock-, EGS^Rpp21^- or siRNA21-treated HeLa cells. Letter P indicates pBluescript background.

Inhibition of Pol I and Pol III transcription is not restricted to knockdown of Rpp29. Thus, knockdown of the subunit Rpp21, which is not shared with RNase MRP, by either siRNA or external guide sequence (Figure 2A)[26] resulted in ∼90% reduction in transcription of the mini-rRNA and 5S rRNA genes (Figures 2B–D). More importantly, nuclear run-on transcription assays confirmed that the level of nascent transcription of endogenous rRNA genes decreased by 5- and 10-fold in cells treated with siRNA21 and EGS^Rpp21^, when compared with control cells (Figure 2E). A more prominent reduction (∼34-fold) in the level of nascent transcription of 5S rRNA gene by Pol III was measured (Figure 2E). Since this nascent transcription reflects the amount of active polymerases on the tested template, the results indicate that RNase P determines the transcription output by Pol I and Pol III.

### Reconstitution assays of Pol I transcription

**Figure 3:**
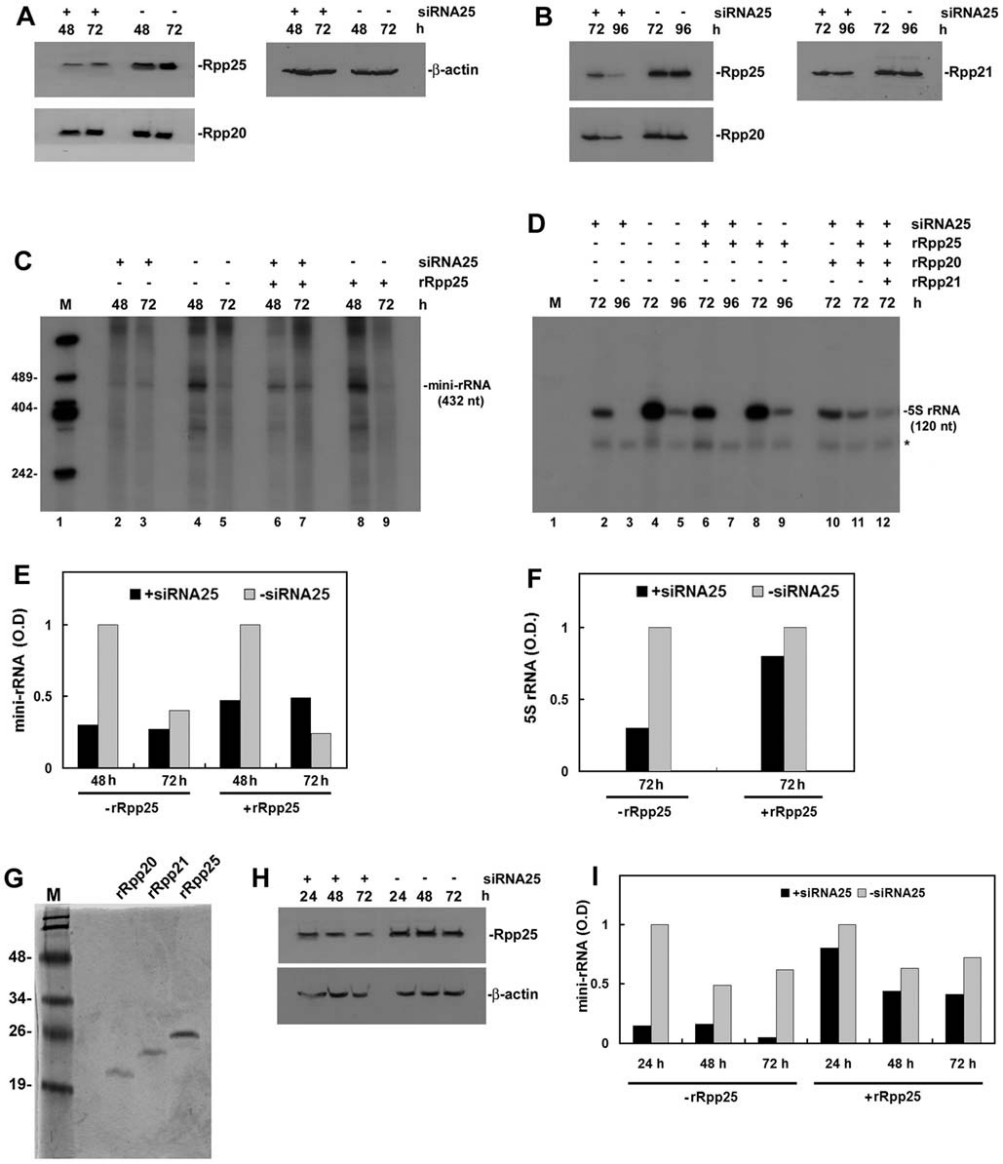
Pol I transcription deficiency can be restored by a recombinant Rpp25 protein. A. HeLa cells were transfected with a plasmid expressing siRNA25 or mock transfected (see Materials and Methods), whole extracts were then prepared from cells at 48 and 72 h after transfection and examined for Rpp25, Rpp20 and β-actin by Western blot analysis. Positions of proteins are depicted. B. Same as in A but transfection was for 72 and 96 h and proteins tested were Rpp25, Rpp20 and Rpp21. C. Reconstitution assays of transcription of the mini-rRNA gene in extracts described in A were performed in the absence or presence of recombinant Rpp25 protein. The resulted 432-nt mini-rRNA was analyzed as in Figure 1C. Exposure time was for 96 h. D. Reconstitution of a human 5S rRNA gene transcription in extracts described in B in the absence or presence of recombinant Rpp25 protein. As controls, recombinant Rpp20 and Rpp21 proteins were used. The resulted 120-nt 5S rRNA was analyzed as in Figure 1C. Asterisk indicates endogenous tRNA labeled with radioactive nucleotide. E. The 432-nt mini-rRNA bands seen in C were quantitated and the optical density (in arbitrary units) was plotted. F. The 120-nt 5S rRNA bands seen in D were quantitated and the optical density (in arbitrary units) was plotted. G. Coomassie blue staining of purified, histidine-tagged rRpp20, rRpp21 and rRpp25 proteins analyzed in 12% SDS/PAGE. Positions of the protein size markers (M) are shown. H. Same as in B but transfection time points were 24, 48 and 72 h. I. Transcription assays of Pol I in the absence and presence of recombinant Rpp25 protein were done as described in C. The 432-nt mini-rRNA band was quantitated and the optical density (in arbitrary units) was plotted. Reconstitution assays described here have been repeated 3 times with different time points and produced similar results.

Knockdown of Rpp21, Rpp29 or Rpp38 by RNA interference has been shown to be accompanied by coordinate inhibition of expression of other protein subunits of human RNase P [26], [27; also data not shown]. This phenomenon made the development of an in vitro reconstitution system for Pol I and Pol III transcription using defined protein components of RNase P unfeasible. However, knockdown of the subunit Rpp25 by siRNA [26] led to moderate inhibition of expression of only two subunits, Rpp20 and Rpp21 (Figures 3A and 3B; and data not shown), while it resulted in severe inhibition of transcription of the mini-rRNA gene (Figure 3C, lanes 2 and 3 versus 4 and 5) and 5S rRNA gene (Figure 3D, lanes 2 and 3 versus 4 and 5). Therefore, we checked if transcription in these extracts can be rescued by a histidine-tagged, recombinant Rpp25 protein that was highly purified through a chelating chromatography column (Figure 3G). Remarkably, the addition of recombinant Rpp25 (see Materials and Methods) to whole extracts obtained from cells with knockdown of Rpp25 resulted in reconstitution of transcription of the mini-rRNA and 5S rRNA genes, when compared with transcription in untreated extracts (Figures 3C and 3D, lanes 6 and 7 versus 2 and 3). The gain-of-function of Pol I transcription was partial (Figure 3E) while that of Pol III transcription was more prominent (Figure 3F; see below). The addition of recombinant Rpp25 to extracts of mock-transfected cells had no comparable consequences on transcription (Figures 3C and 3D, lanes 8 and 9 versus 4 and 5). The addition of recombinant Rpp20 protein (Figure 3G) had no effect on transcription (Figure 3D, lane 10 versus 2), while inclusion of recombinant Rpp20 and Rpp25 or recombinant Rpp20, Rpp21 and Rpp25 proteins rather reduced transcription (Figure 3D, lanes 11 and 12 versus 2). Rpp20 and Rpp25 belong to the Alba-like superfamily of chromatin proteins [28] and it seems that the former protein neutralizes the effect of Rpp25 on transcription.

Flow cytometry analysis revealed a decrease in the proliferation of the cells described above after 2 and 3 days in culture, in which cells reached high confluence (data not shown), and thus exhibited reduced activity of Pol I (Figure 3C, lane 4 versus 5). This reduction in Pol I activity may have counteracted the effect of recombinant Rpp25 in reconstitution assays. Therefore, we repeated the reconstitution experiment as described in Figure 3C but this time we included a 24 h time point, in which knockdown of Rpp25 was evident (Figure 3H). The results show that the addition of recombinant Rpp25 to the extract obtained at 24 h time point brought to almost full recovery of transcription of the mini-rRNA gene, when compared to transcription in extracts obtained at later time points (Figure 3I).

The findings described above provide evidence that a protein subunit of human RNase P can substitute for its endogenous counterpart by reconstituting transcription of Pol I and Pol III.

### Protein subunits and H1 RNA are required for efficient Pol I transcription

**Figure 4:**
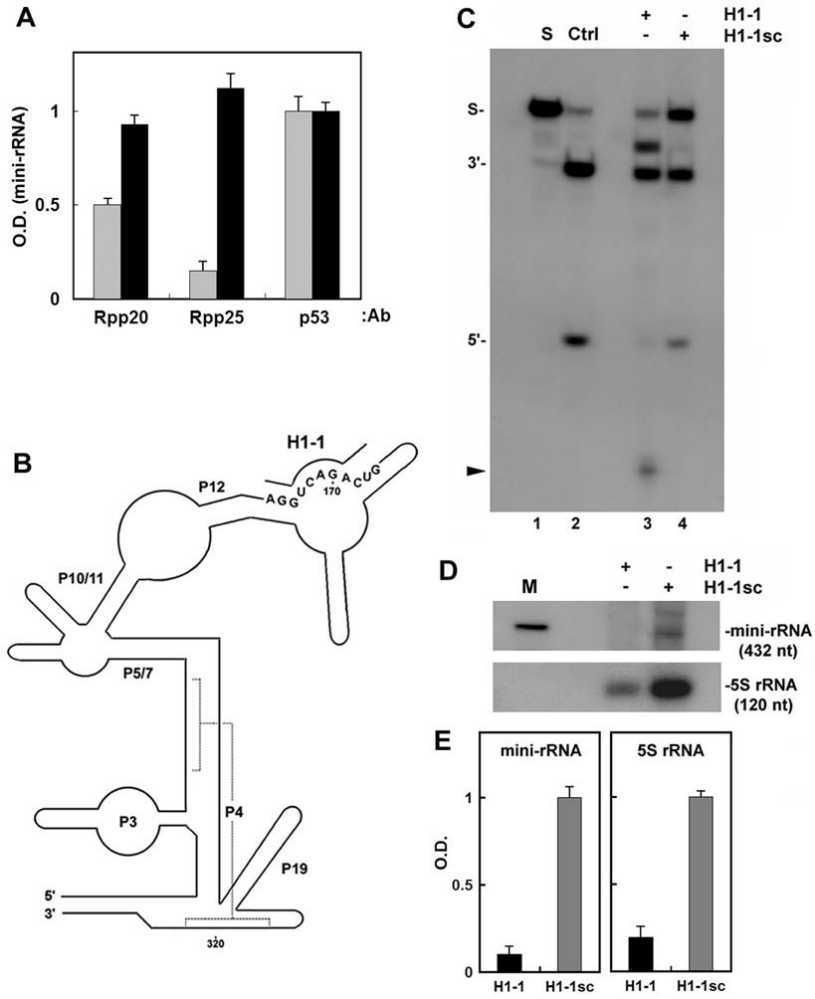
H1 RNA and protein subunits of RNase P are required for Pol I transcription in extracts. A. Whole HeLa extracts were subjected to immunodepletion analysis using 200 µL of serum containing polyclonal rabbit antibodies against Rpp20, Rpp25 or p53. Transcription reactions in the immunodepleted extracts (grey bars) or in extracts reconstituted with their corresponding immunoprecipitates (black bars) were carried out using the mini-rDNA gene and labeled RNAs were analyzed as described in Figure 1C. The 432-nt mini-rRNA band was quantitated and the optical density (in arbitrary units) was plotted. B. A proposed secondary structure of H1 RNA and the nucleotide sequence against which the antisense H1-1 deoxyoligonucleotide was directed. The upper half of H1 RNA represents the specificity domain. Conserved domains, including the P4 pseudoknot in the lower (catalytic) domain are shown. C. Whole HeLa extracts (15 mg/ml) were incubated with 8 µg of H1-1 (lane 3) or scrambled H1-1sc (lane 4) deoxyoligonucleotide in the presence of RNase H for 45 min as described [10]. Extracts were then assayed for RNase P activity in processing of ^32^P-precursor tRNA^Tyr^, and cleavage products were analyzed in an 8% sequencing gel. The 5′ leader sequence (5′) and shorter species (arrow head) generated as a result of substrate miscleavage, are indicated. A concentrated DEAE-purified RNase P preparation (Ctrl; lane 2) was used as control for the correct cleavage of the substrate. D. Whole HeLa extracts described in C were subjected to transcription of the mini-rDNA gene and 5S rRNA genes as described in Figure 1C. E. Optical density of the mini-rRNA and 5S rRNA bands seen in panel D.

Immunodepletion of active RNase P from whole HeLa extracts by the use of polyclonal antibodies directed against Rpp20 and Rpp25 (see Materials and Methods)[10] resulted in reductions of ∼40% and 80% in transcription of the mini-rRNA gene, which could be restored by adding back their corresponding immunoprecipitates (Figure 4A). Moreover, targeted cleavage of the H1 RNA moiety in whole HeLa extracts by RNase H and an antisense deoxyoligonucleotide H1-1, which is directed against the specificity domain of H1 RNA (Figure 4B), caused incorrect processing of the 5′ leader sequence of precursor tRNA^Tyr^ by RNase P (Figure 4C, lane 3 versus 2; arrow head) and abolished transcription of the mini-rDNA gene (Figures 4D and 4E). By contrast, the use of a scrambled H1-1 oligonucleotide, H1-1sc, did not alter enzyme specificity (Figure 4C, lane 4 versus 2) or transcription (Figures 4D and 4E). Similar results were obtained for transcription of the 5S rRNA gene (Figures 4D and 4E). Neither RNase H nor oligonucleotides alone had an effect on RNase P or transcription (data not shown; ref. 10). Since H1 RNA discriminates RNase P from RNase MRP, the results establish that the former ribonucleoprotein, containing an intact H1 RNA, is implicated in transcription by Pol I.

### RNase P copurifies and associates with components of Pol I and its transcription factors

**Figure 5:**
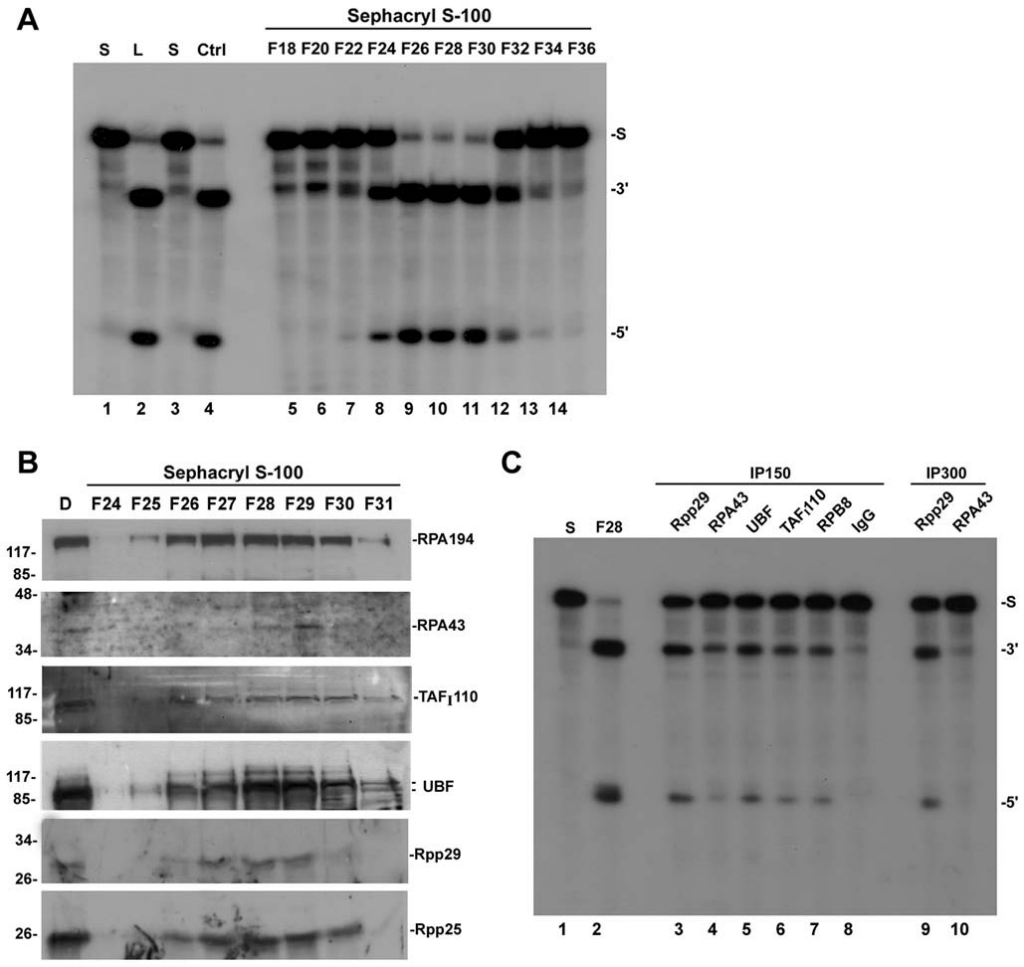
Pol I components copurify and associate with human RNase P. A. A DEAE-purified RNase P was loaded (L, load) into a Sephacryl S-100 gel filtration column and eluted fractions were assayed for RNase P activity in processing of ^32^P-labeled precursor tRNA^Tyr^ (S) to mature tRNA (3′) and 5′ leader sequence (5′). Substrate cleavage was analyzed as in Figure 4C with control HeLa RNase P (Ctrl). B. Western blot analysis of Sephacryl S-100 eluted fractions using antibodies against RPA194, RPA43, TAF_I_110, UBF, Rpp29 and Rpp25. Preparations of DEAE-purified RNase P (first lane) was used as control for input. Proteins were separated in 12% SDS/PAGE and the positions of the corresponding proteins and the protein size markers are shown. C. Fraction F28 described in A was subjected to immunoprecipitation analysis using antibodies against Rpp29 (lane 3), RPA43 (lane 4), UBF (lane 5), TAF_I_110 (lane 6), RPB8 (lane 7) and IgG (lane 8) in the presence of 150 mM KCl (IP150). For Rpp29 and RPA43 immunoprecipitation was also performed at 300 mM KCl (lanes 9 and 10). Immunoprecipitates obtained were tested for RNase P activity in processing of a ^32^P-labeled precursor tRNA^Tyr^ (S). RNase P in fraction F28 was tested as control for enzyme activity (lane 2) and cleavage products were analyzed as in A.

To check if RNase P is associated with Pol I, S100 extracts of HeLa cells were fractionated in a DEAE-Sepharose anion exchange chromatography column [13; see Materials and Methods] and the eluted fractions with the peak of RNase P activity were then purified in a hydrophilic Sephacryl S-100 gel filtration column [14]. The excluded fractions were assayed for RNase P activity in tRNA processing (Figure 5A) and tested for the presence of Rpp29 and Rpp25 (Figure 5B; lower panels). Components of Pol I, such as RPA194 and RPA43, and its associated transcription factors, including the upstream transcription factor UBF and the TBP-associated factor TAF_I_110, were all detected in fractions F26-F30 enriched with RNase P (Figure 5B). Moreover, active RNase P is physically associated with RPA43, RPB8, UBF and TAF_I_110 in these fractions as revealed by coimmunoprecipitation experiments (Figure 5C, lanes 4–7).

The above findings demonstrate that an active RNase P associates with components of Pol I, which seems to exist in a large complex with its transcription initiation factors. The purification of a large (M_r_>2000 kDa) murine Pol I holoenzyme with its transcription factors has been reported [29].

### RNase P and Pol I are located at the promoter and coding region of rDNA

**Figure 6:**
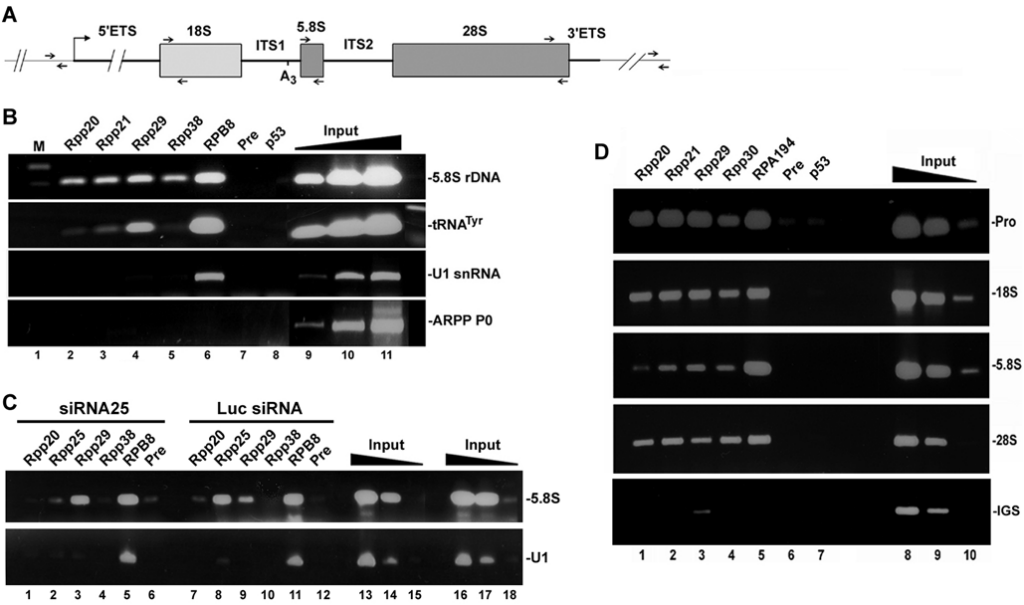
Protein subunits of RNase P occupy the promoter and coding region of rDNA. A. Structure of a rRNA gene, showing 18S, 5.8S and 28S rRNA flanked by 5′ETS and 3′ETS and intervened by ITS1 and ITS2. RNase MRP cleaves at the A3 site in the ITS1. Small arrows indicate locations of primers used for PCR. B. ChIP was performed with rapidly dividing HeLa cells using affinity-purified polyclonal antibodies against Rpp20 (lane 2), Rpp21 (lane 3), Rpp29 (lane 4), Rpp38 (lane 5), RPB8 (lane 6), preimmune serum (lane 7) or p53 (lane 8). DNA of the precipitated chromatin was analyzed by PCR for the presence of 5.8S rDNA, tRNA^Tyr^, U1 snRNA and ARPP P0 genes. Positive PCR products for each gene using 0.01, 0.1 and 1% of input DNA are shown (lanes 9–11). This panel is a composite of two mini-agarose gels with the same exposure time. C. HeLa cells were transfected with siRNA25 (lanes 1–6), which caused marked knockdown of Rpp25 (data not shown), or with control luciferase siRNA (lanes 7–12) for 48 h. ChIP analysis was then performed for 5.8S rDNA and U1 snRNA gene using antibodies against Rpp20, Rpp25, Rpp29, Rpp38, RPB8 and preimmune serum. Positive PCR reactions using 0.01, 0.1 and 1% of input DNA are shown (lanes 13–18). D. ChIP analysis was performed with cells as in B using antibodies against Rpp20 (lane 1), Rpp21 (lane 2), Rpp29 (lane 3), Rpp30 (lane 4), RPA194 (lane 5), preimmune serum (lane 6) or p53 (lane 7). DNA of the precipitated chromatin was analyzed by PCR for the presence of the promoter, 18S, 5.8S, 28S or IGS DNA using the corresponding primers shown in A. Positive PCR reactions for each rDNA region using 0.01, 0.1 and 1% of input DNA are shown (lanes 8–10).

To have a mechanistic explanation of how human RNase P acts with Pol I in rRNA gene transcription, we tested if the former multi-protein complex associates with rDNA in rapidly dividing HeLa cells by chromatin immunoprecipitation (ChIP) analysis (see Materials and Methods)[10]. Genes for rRNA are organized in tandem transcription units separated by intergenic spacers [30], [31] and each unit has 18S, 5.8S and 28S rRNA coding regions that are interspersed by internal and external transcribed spacers (Figure 6A). ChIP analysis revealed 5.8S rDNA in chromatin samples brought down by antibodies directed against Rpp20, Rpp21 (not shared with RNase MRP), Rpp29 and Rpp38 (Figure 6B, lanes 2–5, respectively), as well as RPB8 (Figure 6B, lane 6), a core protein component of Pol I (also of Pol II and III). No PCR product was seen when preimmune rabbit serum or antibody directed against the tumor suppressor p53 were tested (Figure 6B, lanes 7 and 8). A human tRNA^Tyr^ gene was detected in chromatin samples brought down by antibodies against Rpp20, Rpp21 and Rpp29 (Figure 6B, lanes 2–4) but not Rpp38 (Figure 6B, lane 5), consistent with previous findings [10]. The U1 snRNA gene, which is transcribed by Pol II, was enriched in chromatin precipitated by the antibody against RPB8 (Figure 6B, lane 6), while the transcriptionally inactive ARPP P0 gene was not detected in any chromatin sample (Figure 6B, lanes 2–8). Chromatin occupancy of 5.8S rDNA by Rpp25 was almost eliminated in cycling HeLa cells transfected with siRNA25 for 48 h, when compared with cells treated with luciferase siRNA (Figure 6C, lane 2 versus 8), while that of Rpp20, Rpp29 and RPB8 showed no comparable decline (Figure 6C). Hence, binding of Rpp25 to rDNA loci is independent of that of other protein subunits of RNase P and Pol I.

We next scanned the human rDNA transcription units (each is ∼14 Kbp) for binding of protein subunits of RNase P by ChIP analysis using specific primers that correspond to distant regions within these units (see Figure 6A). In these experiments, the genomic DNA was sheared to small pieces of ∼500 bp to allow high resolution of chromatin binding regions (data not shown). Surprisingly, we found that Rpp20, Rpp21, Rpp29 and Rpp30 associated with the promoter region, in addition to the coding region spanning 18S rDNA and 28S rDNA (Figure 6D, lanes 1–4). Similarly, RPA194, a protein subunit of Pol I, bound to the promoter and coding region of rDNA (Figure 6D, lane 5), consistent with previous findings reported by others [32]. RPA194 did not bind to the intergenic sequence located ∼5 Kbp downstream of the terminator region of rDNA, which was also the case with the protein subunit of RNase P, except for Rpp29 that showed weak signal of binding (Figure 6D, lower panel). This latter finding rules out the possibility that RNase P is distributed uniformly on rDNA repeats.

### Dissociation and reassociation of RNase P with chromatin of rDNA during the cell cycle

**Figure 7:**
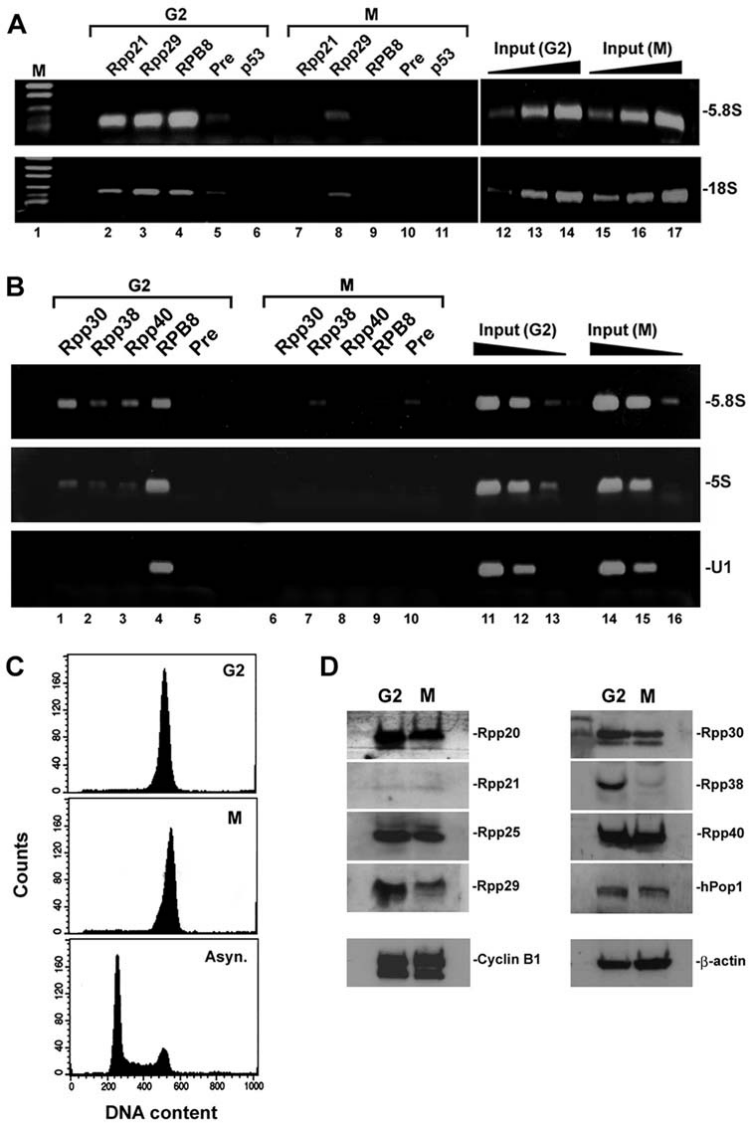
Protein subunits of RNase P dissociate from rDNA loci in mitotic cells. A. HeLa cells were arrested at G2/M by treatment with nocodazole for 16 h. Mitotic cells were separated from adhered G2 cells as described [35]. ChIP was performed with G2 (G2) and mitotic (M) cells using antibodies against Rpp21 (lanes 2 and 7), Rpp29 (lanes 3 and 8), RPB8 (lanes 4 and 9), preimmune serum (lanes 5 and 10) or p53 (lanes 6 and 11). Detection of 5.8S rDNA and 18S rDNA was done as in Figure 6D. Positive PCR reactions using 0.01, 0.1 and 1% of input DNA are shown (lanes 12–17). The PCR signal of Rpp29 seen in mitotic cells (lane 8) was not seen in two other independent experiments (data not shown) and hence its presence may be related to incomplete separation of M and G2 cells. B. As in A but Rpp30, Rpp38, Rpp40, RPB8 and preimmune serum were tested in ChIP. The U1 snRNA gene that is transcribed by Pol II was tested as control. C. FACS analysis of cells described in C. Asynchronous HeLa cells were used as control. D. Western blot analysis of whole extracts prepared from G2 and M cells described in C by using affinity-purified antibodies directed against the indicated protein subunits of human RNase P. Rpp14 and hPop5 were difficult to detect (data not shown). Mitotic cyclin B1 and β-actin were analyzed as control.

Transcription of rRNA genes is regulated during the cell cycle, whereby it gradually increases from G1 to S phase, peaks at G2 and ceases at mitosis [33], [34]. To show that RNase P associates with transcriptionally active rRNA genes, HeLa cells were synchronized to G2/M by treatment with nocodazole, a microtubules depolymerization inhibitor, and then the separated G2 and M cell populations [35; see Materials and Methods] were subjected to flow cytometry (Figure 7C) and ChIP analyses. Rpp21 and Rpp29 were associated with 5.8S rDNA and 18S rDNA in G2 cells but fully or partially disengaged from these loci in mitotic cells (Figure 7A, lanes 2 and 3 versus 7 and 8, respectively). Moreover, chromatin occupancy of these pre-rRNA coding regions by RPB8, which represents Pol I, was evident in cells at G2 but not at M phase (Figure 7A, lane 4 versus 9), an observation that is consistent with cessation of transcription [34], [36]. In fact, all protein subunits of RNase P, including Rpp30, Rpp38 and Rpp40, were bound to rDNA in G2 cells but not in mitotic cells (Figure 7B, lanes 1–3 versus 6–8 and data not shown). Western blot analysis showed that the expression of some protein subunits, such as Rpp29 and Rpp38, decreased in mitotic cells, when compared with G2 cells, while that of Rpp20 and Rpp40 remained largely unchanged (Figure 7D; see below). This implies that the detachment of these protein subunits from rDNA in mitosis is a shared property but their fate is not interrelated (see below).

**Figure 8:**
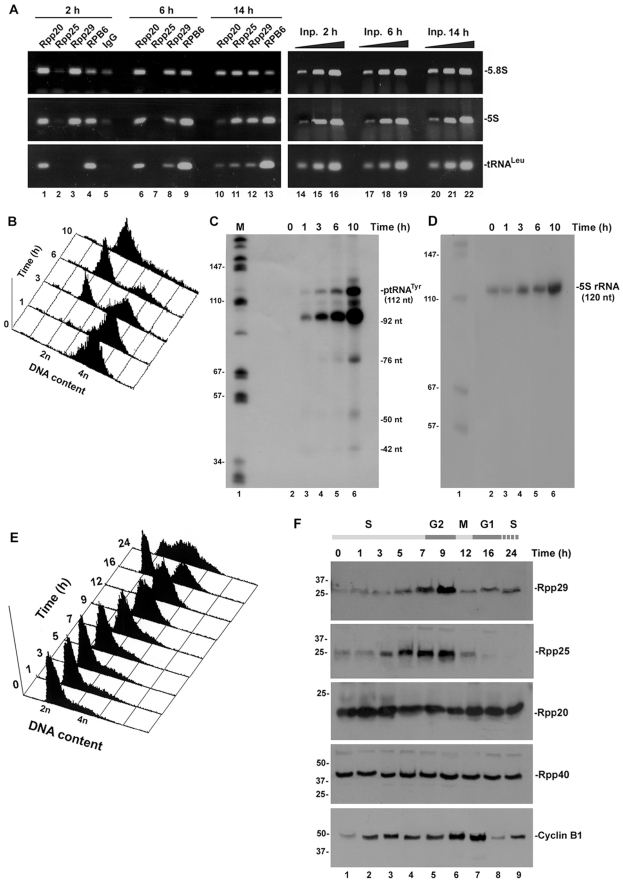
Reassociation of protein subunits of RNase P with chromatin of target genes. A. HeLa cells were synchronized by nocodazole for 16 h before mitotic cells were separated from adhered G2 cells. Mitotic cells were released from the inhibitor for 2, 6 and 14 h. Cells were at early G1, mid G1 and S phase as determined by flow cytometry analysis (data not shown). ChIP was then performed with synchronized cells using antibodies against Rpp20 (lanes 1, 6 and 10), Rpp25 (lanes 2, 7 and 11), Rpp29 (lanes 3, 8 and 12), RPB6 (lanes 4, 9 and 13) or control IgG antibody (lane 5). DNA extracted from the precipitated chromatin was analyzed by PCR for the presence of 5.8S rDNA, 5S rDNA and tRNA^Leu^ genes. Linear PCR reactions using 0.01, 0.1 and 1% of input DNA are shown (lanes 14–22). B. HeLa cells were synchronized as described in A and at the indicated time points after release from nocodazole they were subjected to FACS analysis. C. Whole extracts obtained from HeLa cells described in B were examined for transcription of a human tRNA^Tyr^ gene as previously described [10]. The resulted primary transcript is 112 nt in length and has an intron that is promptly excised to release the exons with 5′ leader and 3′ trailer sequences (42 and 50 nt in length, respectively). The exons are ligated to produce a 92-nt intermediate precursor tRNA^Tyr^, which is processed by RNase P to remove the 5′ leader sequence (5 nt) and by a 3′ endonuclease that eliminates the 3′ trailer sequence (11 nt) to generate a mature 76-nt tRNA^Tyr^. D. Whole HeLa extracts described in B were assayed for 5S rRNA gene transcription. E. HeLa cells were synchronized to G1/S phase by treatment with hydroxyurea for 16 h. Cells were then released from inhibitor and analyzed by FACS for their DNA content at the indicated time points. Cells were at mitosis at 12 h. F. Western blot analysis of whole extracts obtained from cells described in E using antibodies against Rpp29, Rpp25, Rpp20 and Rpp40 and cyclin B1. Cell cycle progression shown on the top was deduced from the FACS data shown in E and from the levels of cyclin B1. Similar results were obtained in three independent synchronization experiments.

We next investigated the recruitment of protein subunits of RNase P after mitosis in synchronized HeLa cells. Strikingly, we found that Rpp20 and Rpp29 reassociated with 5.8S rDNA after 2 h of exit of synchronized HeLa cells from mitosis (Figure 8A, lanes 1 and 3) but Rpp25 did not (Figure 8A, lane 2). Similar pattern of chromatin occupancy by these three subunits was observed when the 5S rRNA gene was analyzed (Figure 8A, lanes 1–3; middle panel), while both Rpp29 and Rpp25 were not detected at the tRNA^Leu^ gene until mid G1 (Figure 8A, lanes 2 and 3 versus 8 and 11). Therefore, recruitment of Rpp20, Rpp25 and Rpp29 to chromatin is not interdependent. Of note, RPB6 promptly bound to 5.8S rDNA, 5S rRNA and tRNA^Leu^ genes at early G1 (Figure 8A, lanes 4, 9 and 13) even though this binding does not reflect vigorous Pol I transcription, since it has been shown that UBF is inactivated at early G1 [33], [34]. Indeed, transcription of human tRNA^Tyr^ and 5S rRNA genes was also impaired at early G1 (Figures 8C and 8D, lane 2 versus 3) but gradually increased as the synchronized cells (Figure 8B) progressed to S phase (Figures 8C and 8D, lanes 3–6). By contrast, binding of Rpp20 to chromatin of 5S rRNA and tRNA^Leu^ genes somewhat decreased at S phase (Figure 8A, lane 10 versus 1 and 6)

The aforementioned observations raised the question if the biosynthesis of the protein subunits of human RNase P during the cell cycle correlates with chromatin binding by RNase P and with the transcriptional activity of Pol I and Pol III. Thus, the steady state levels of several protein subunits of RNase P were determined in synchronized HeLa cells that were released from hydroxyurea, which arrests cells at G1/S phase (Figure 8E). Expression of Rpp25 and Rpp29 increased during the S phase, peaked at G2, declined at mitosis and recovered at G1 (Figure 8F, lanes 1–9). This pattern agrees with the reported activity of Pol I and Pol III during the cell cycle [34], [35]. However, we found that the expression of Rpp20 and Rpp40 rather remained largely unchanged throughout the phases of the cell cycle, including mitosis (Figure 8F, lanes 1–9), in which these subunits also detached from chromatin (data not shown). Hence, the biosynthesis of these latter protein subunits does not reflect their dynamic binding to chromatin or transcription during the cell cycle.

Taken together, the results described above indicate that restoration of Pol I and Pol III transcription after mitosis is not correlated with the recruitment of a fully preassembled RNase P on rDNA and small noncoding RNA genes. To the contrary, its assembly involves a dynamic and stepwise association process, which includes two Alba-like chromatin proteins, Rpp20 and Rpp25.

## Discussion

We have shown that human RNase P is required for efficient transcription of rDNA by Pol I. Thus, inactivation of RNase P by targeting its protein and RNA subunits for destruction inhibits rRNA gene transcription by Pol I, an indication that this complex acts in the form of ribonucleoprotein. RNase P associates with components of Pol I and its transcription factors and affects nascent transcription by Pol I, as well as Pol III. RNase P binds to chromatin of the promoter and coding region of rRNA genes. Chromatin occupancy by RNase P is dependent on active transcription of rRNA genes and is linked to the cell cycle. Combined with the role of RNase P in Pol III transcription and binding to chromatin of 5S rRNA and tRNA genes [10], [11], the data presented in this study suggest that this chromatin-associated complex is critical for the coordinate regulation of ribosome biogenesis and protein translation.

### A novel role for RNase P in the nucleolus

In *S. cerevisiae*, the majority of the RNase P RNA exists in the nucleolus, in which clustering of tRNA genes and processing of precursor tRNAs take place [37]. In situ RNA hybridization analysis also visualized H1 RNA in the nucleolus, thus raising the possibility that RNase P may be functional in ribosome biogenesis. Our study establishes that a functional human RNase P exists in the nucleolus and that it is implicated in transcription of rRNA genes. ChIP analysis demonstrates that many protein subunits of RNase P, including its subunit Rpp21 that is not shared with RNase MRP, bind to chromatin of transcriptionally active rDNA loci. This nucleolar form of RNase P is comparable, in terms of its protein composition, to that initially characterized as a tRNA processing holoenzyme [13].

In yeast, processing of rRNA is coordinated in a transcription-dependent manner [38]–[40]. Yeast Pol I associates with a nucleolar substructure that is active in the synthesis and processing of rRNA [41] and it has been demonstrated that transcription and processing of rRNA are coordinated through specific components of the small ribosomal subunit processome [38], [40], [42]. However, human RNase P does not act on transcription of rDNA as a processing factor. First, the mini-rRNA gene construct was designed to have a rDNA promoter fused to adjacent 5′ETS segment, which has no known cleavage sites that could recruit processing factors at the transcript level. Nonetheless, transcription driven by this promoter is inhibited in extracts lacking functional RNase P, as a result of depletion of its subunits, including H1 RNA and Rpp21 that are not shared with RNase MRP. In fact, it has been shown that transcription and processing factors are recruited separately in mammalian cells [39], [43]. Second, RNase P binds to the promoter region of rDNA and associates with components of the transcription initiation machinery, e. g. Pol I, UBF and SL1 complex, suggesting that it is involved in transcription initiation. Third, RT-PCR analysis of 5′ETS unveils a substantial reduction in pre-rRNA synthesis in cells having inactive RNase P. 5′ETS is known to be processed rapidly and cotranscriptionally from the primary transcript of rRNA and thus reflects transcription initiation by Pol I.

### Assembly of a chromatin-associated RNase P and its link to the cell cycle

Binding of RNase P to chromatin of rDNA is dynamic in the sense that it is linked to transcription and the cell cycle. In mitosis, RNase P detaches from chromatin of rDNA, which concurs with cessation of transcription, while most of the protein subunits of RNase P were found to be associated with chromatin of rDNA at G2 phase, in which transcription by Pol I is elevated. At early G1, however, not all the protein subunits of RNase P are equally reassociated with chromatin of rDNA. For example, Rpp20 and Rpp29 but not Rpp25 are promptly recruited to rDNA after exit from mitosis, while Rpp25 binds to rDNA loci at the S phase (or late G1), which coincides with increased transcription by Pol I. Accordingly, Rpp25 seems to contribute to the elevated activity of Pol I at S phase (or late G1), a conclusion that is supported by the finding that recombinant Rpp25 stimulates transcription by Pol I in whole cell extracts lacking endogenous Rpp25. Similarly, recruitment of RNase P to 5S rRNA and tRNA genes after mitosis proceeds via a dynamic and stepwise association process of its subunits.

A line of evidence supports the prospect that protein subunits of human RNase P could be recruited as independent entities to chromatin of target genes. Thus, RNA interference shows that knockdown of one protein subunit does not necessarily obstruct the recruitment of other subunits on target genes in cycling cells, even though transcription is inhibited. For example, knockdown of Rpp25 did not lead to disengagement of Rpp20 and Rpp29 from rDNA (Figure 6C) and knockdown of Rpp29 did not affect chromatin occupancy of tRNA and 5S rRNA genes by Rpp21 [10]. Moreover, Rpp21 and Rpp29 differentially disengage from chromatin of tRNA and 5S rRNA genes in response to cessation of proliferation of cells [10]. Another research group showed that Rpp29 rapidly shuttles between the nucleoplasm and nucleolus, suggesting that this core component of RNase P is highly dynamic and not permanently bound to a fixed RNase P [44]. That RNase P is recruited as individual subunits (or partial sub-complexes) is consistent with the finding that subunits of Pol I rapidly and massively enter the nucleolus as distinct subunits rather than as part of a preassembled holoenzyme [45] and that the assembly of Pol I proceeds via a sequential manner in each transcription cycle [39], [45]. More importantly, it has been shown that the recruitment efficiency and retention of Pol I components at the promoter region of rDNA is used to control the transcriptional output of rRNA genes [46]. Apparently, these latter findings contrast the existence of assembled holoenzymes of Pol I [29] and RNase P [13]. Nonetheless, the purified Pol I-RNase P complex described in this study may constitute the fraction of these holoenzymes that have already been assembled and/or engaged in transcription. Nuclear run-on transcription assays indicate that the rate of nascent transcription of rDNA and 5S rRNA genes, which reflects the number of active Pol I and Pol III on these genes, is reduced in cells with inactivated RNase P. Thus, RNase P determines the output of transcription by active polymerases. Remarkably, ChIP analysis showed that chromatin occupancy by Pol I and Pol III (as represented by the core components RPB6 and RPB8) did not change perceptibly in cells with inactivated RNase P (Figure 6C; also refs. 10 and 11), an indication that these two polymerases can be recruited to their corresponding templates, even though there is no active transcription. Accordingly, RNase P defines those polymerases that are engaged in active transcription. However, the exact role of RNase P that is engaged in transcription remains unknown. This complex can act as auxiliary transcription factor, chromatin modifier or as new type of chromatin remodeling complex. In the latter case, it has been shown that RNase P has an ATPase activity embedded within its Rpp20 subunit [47] but this chromatin-associated subunit is not related to the SNF2-like ATPases. Nonetheless, binding of RNase P to the entire rDNA transcription unit (Figure 6D) could also suggest that this complex has multiple roles in the various steps of transcription by Pol I.

Finally, binding of a large ribonucleoprotein complex, such as human RNase P with its two Alba-like proteins, Rpp20 and Rpp25, to chromatin of hundreds of genes transcribed by Pol I and Pol III should have global effects on the structure, spatial organization and function of the genome [48]. As RNA can act as a scaffold, recruiting factor or sequence-specificity factor in the modification and function of chromatin [49], elucidation of any potential role of H1 RNA of RNase P in this regard is of particular interest.

## Materials and Methods

### Cell transfection, synchronization and flow cytometry analysis

Adherent HeLa cells were grown in high glucose DMEM (Invitrogen) supplemented with 5% fetal bovine serum, streptomycin (100 µg/ml), penicillin (100 U/ml) and Nystatin (12.5 U/ml). Cells were incubated in 5% CO_2_ at 37°C. For transfection, 1–5×10^5^ cells grown in 92×17 mm style petri dishes were transfected with the desired siRNA (15–30 µg/dish) or plasmid DNA (15 µg) in 10 ml medium using the calcium phosphate method. For knockdown of Rpp21 and Rpp25, plasmids (10–15 µg) carrying siRNA-coding genes (kind gifts of Prof. Sidney Altman)[26] were introduced into cells.

For synchronization, HeLa cells were grown in 92×17 mm style petri dishes for 50% confluence and then treated with nocodazole (40 ng/ml) for 16 h. The dishes were shaken to detach mitotic cells from G2-enriched, adhered cells as described by White et al. 1995 [ref. 35]. Synchronization of cells by hydroxyurea (2.5 mM) was for 16 h. Cells were released from the inhibitor by replacing the medium with warm, fresh one. After time points indicated in each experiment the cells were then stained with propidium iodide for DNA content analysis by fluorescence-activated cell sorter. Whole cell extracts were prepared as described [10]. Protein concentrations in extracts were 10–15 mg/ml.

### RT-PCR analysis

Reverse transcription was performed using M-MLV reverse transcriptase (Promega) with equal amounts of total RNA (1–2 µg) extracted from treated HeLa cells and 5–10 pmol of gene-specific reverse primers (see below). Amplification of the 5′ETS sequence of the reverse-transcribed pre-rRNA by PCR was performed as described by others [50] except that the reaction contained 5% Dimethyl sulfoxide (DMSO). Reverse transcription and PCR amplification of U1 snRNA was done using primers described previously [10].

### Purification and analysis of human RNase P

Rapidly dividing HeLa cells (at ∼50% confluence) in sixty 175-cm square flasks were pelleted, disrupted, and the cell homogenate was centrifuged at 7,000 rpm followed by another centrifugation at 31,000 rpm in a Beckman Ti60 rotor to obtain S100 crude extract [13]. This S100 extract was loaded on a DEAE-Sepharose anion exchange chromatography column equilibrated with buffer A that contains 10 mM Tris-HCl, pH 8.0, 100 mM KCl, 2.5 mM MgCl_2_ and 1 mM DTT. RNase P was then eluted from the column using a 100–500 mM KCl gradient [13]. RNase P activity in the eluted fractions was examined by processing of the 5′ leader of a ^32^P-labeled E. coli precursor tRNA^Tyr^. RNase P activity is eluted at 250–300 mM KCl [13], and therefore it coelutes with Pol I [51]. However, these fractions enriched with RNase P and Pol I are inactive in transcription of a mini-rRNA gene (data not shown), most likely because TIF-IA dissociates from Pol I in the presence of MgCl_2_ [51], which was included in buffer A. Fractions enriched with active RNase P were then pooled, concentrated and fractionated in Sephacryl S-100 HR chromatography column with protein size markers [14]. Volumetric flow rate of the gel filtration column was set at ∼0.8 ml/min. The eluted fractions were assayed for the presence of active RNase P as described above. RNase P eluted as a large particle from the column (with fractionation range of M_r_ = 1×10^5^) in the void volume.

### In vitro transcription of Pol I and Pol III and reconstitution assays

In vitro transcription reactions of the mini-rRNA gene in newly prepared whole HeLa extracts were performed in a final volume of 25 µl that contained 15 µl of extract, 1× transcription buffer (12 mM Tris-HCl at pH 7.9, 5 mM MgCl_2_, 80 mM KCl, 0.5 mM DTT, 10 mM creatine phosphate), NTPs (0.66 mM ATP, 0.66 mM CTP, 0.66 mM GTP, 0.01 mM UTP), 5 µCi of [α-^32^P]-UTP (3000 Ci/mmol; Amersham) and 0.25 µg of plasmid carrying the mini-rDNA gene. After 1 h of incubation at 30°C, reaction mixtures were diluted 1∶1 with H_2_O, passed through a G-50 column, diluted to 250 µl with 1× digestion buffer (20 mM Tris-HCl, pH 7.9, 250 mM sodium acetate, 1 mM EDTA, 0.25% SDS), and digested with 120 µg/ml Proteinase K for 30 min at 37°C. RNA was recovered following phenol∶chloroform∶isoamylalcohol extraction by ethanol precipitation and labeled RNAs were analyzed in 8% polyacrylamide gels. Bands were visualized by autoradiography and quantitated by Scion Image software.

Transcription reactions with human 5S rRNA and tRNA^Tyr^ genes were performed as previously described [10].

For reconstitution assays, recombinant proteins purified through His-bind affinity chromatography columns [13] in folded state were dialyzed against excess volumes of 1× transcription buffer. The purity of the recombinant proteins were >95% as determined by SDS/PAGE followed by coomassie blue staining. Recombinant proteins at optimal concentration, 0.4 µg per 15 µl of a newly prepared whole HeLa extract (∼200 µg protein), were then added to the extracts for 30 min on ice before the start of the transcription reactions as specified above for each gene.

### Nuclear run-on transcription assay

This assay was essentially performed as previously described by Prieto and McStay 2007 [43]. Nuclei were treated with DNase and Proteinase K and labeled RNA was isolated by phenol∶chloroform∶isoamylalcohol extraction and was then hybridized to probes immobilized on dot blots. The probes were pBluescript carrying human 5′ETS fragment of rDNA and 5S rRNA gene.

### Western blot analysis and immunoprecipitation assays

Whole cell extracts or protein eluates from chromatography columns were separated in 12% polyacrylamide/0.1% SDS gels. Proteins were electrotransferred to nitrocellulose filters and immunoblotted with primary antibodies in 1∶100–400 dilution. A 1∶28,000 dilution of the corresponding secondary antibody was used. Blots were washed and bands were visualized using the ECL chemiluminescent kit, following instructions of the manufacturer (Amersham).

RNase P from whole HeLa extracts was immunoprecipitated with polyclonal anti-Rpp antibodies or with monoclonal and polyclonal antibodies directed against Pol I, TAF_I_110, UBF, as described [10]. Immunoprecipitates were assayed for RNase P activity in processing of the 5′ leader sequence of a ^32^P-labeled precursor tRNA^Tyr^ in 1× PA buffer mixed with 1× TNET buffer containing 2 mM Tris-HCl, pH 7.5, 35 mM NaCl, 0.1 mM Na_2_EDTA, 1 mM 2-mercaptoethanol, 0.01% Triton X-100, 4 U of rRNasin and 12 µg Poly I∶C. Cleavage products were separated in 8% polyacrylamide/7 M urea gel.

### Chromatin immunoprecipitation analysis

ChIP analysis was done essentially as previously described [10]. Asynchronized or synchronized HeLa cells were collected, washed with 1× PBS, and cross-linked with 0.5% NP-40/1× PBS containing 1% formaldehyde for 10 min at 37°C. Cells were rinsed with ice-cold 0.5% NP-40/1× PBS and incubated for 30 min in high-salt buffer (1 M NaCl, 0.5% NP-40, 1× PBS). Cells were collected, washed with 0.5% NP-40/1× PBS, and then resuspended in low-salt buffer (0.1 M NaCl, 0.1% NP-40, 10 mM Tris-HCl at pH 8.0, 1 mM EDTA). After 30 min, cells were centrifuged at 480×g for 10 min and subjected to 10 strokes through a 23-gauge needle. Nuclei obtained after centrifugation were resuspended in low-salt buffer containing 2% sarkosyl and transferred to a sucrose cushion (0.1 M sucrose, 0.1 M NaCl, 10 mM Tris-HCl at pH 8.0, 1 mM EDTA, 0.5% NP-40), and then spun for 10 min at 4000×g. The pellet was resuspended in TE (10 mM Tris-HCl at pH 8.0, 1 mM EDTA) and spun again, and genomic DNA was sheared by sonication (20×30 sec, duty cycle 80%) to produce stretches of chromatin of ∼1000 base pairs (bp) in length. In the experiment described in Figure 6D, the genomic DNA was cut off to smaller pieces of ∼500 bp. The sonicated material was diluted in 1/10 volume of ×11 NET buffer (1.65 M NaCl, 550 mM Tris-HCl at pH 7.4, 5.5 mM EDTA, 5.5% NP-40).

Sonicated material (0.2 ml per each immunoprecipitation) was precleared for 30 min with 20 µl of Protein A/G Plus agarose beads (Santa Cruz Biotechnology) and then blocked with 2 µg of salmon sperm (Invitrogen). After centrifugation, the sample was subjected to IP overnight at 4°C using a nutating device with the appropriate antibody coupled to beads (coupling was for 6 h at 4°C) and in the presence of 4 µg of salmon sperm. Precipitated complexes were washed three times with 1 ml of RIPA buffer (50 mM Tris-HCl at pH 8.0, 150 mM NaCl, 0.1% SDS, 0.5% deoxycholate, 1% NP-40), three times with 1 ml of LiCl buffer (10 mM Tris-HCl at pH 8.0, 250 mM LiCl, 1 mM EDTA 0.5% deoxycholate, 0.5% NP-40), and three times with 1 ml of TE. Immunoprecipitated material was eluted twice with 200 µl of 1% SDS/TE and incubated overnight at 42°C in 0.4 ml volume of elution buffer containing 120 µg/ml Proteinase K. DNA was extracted twice with phenol/chloroform/isoamyl alcohol (25∶24∶1 v/v), ethanol-precipitated, washed with 70% ethanol, dried, and resuspended in 25 µL distilled water for use in PCR analysis.

Each PCR reaction contained 2.5 µL of each primer (final concentration of 1 µM), 5 µl of 10× reaction mixture, and 4 µl of DNA for each IP sample. Input DNA was diluted in distilled water before PCR. PCR amplification programs and primer sequences for 5S rRNA, tRNA^Tyr^, U1 snRNA, ARPP P0 were described [10]. Primers for promoter rDNA were described in Philimonenko et al. 2004 [32]. Primers for 5.8S rDNA were 5′- CGACTCTTAGCGGTGGATCAC-3′ and 5′-AAGCGACGCTCAGACAGGCGT-3′, for 18S rDNA: 5′-CCTTTAACGAGGATCCATTGGA-3′ and 5′-GACACTCAGCTAAGA GCATCGAG -3′, for 28S rDNA: 5′-CTCTTCCTATCATTGTGAAGCAG-3′ and 5′-CAAATGTCTGAACCTGCGGTTC-3′ and for IGS: 5′-TGTTCTTGGGGGTGGGTTGAC-3′ and 5′-GAAGAGGTTCCGATGGGAAGTTG-3′.

### RNase H digestion assay

Targeting the H1 RNA for specific cleavage by RNase H has been previously described [10]. Whole HeLa extract (15 µl; 10–15 mg/ml; not diluted) was incubated with 8 µg of H1-1 or scrambled H1-1sc deoxyoligonucleotide (Sigma, Israel) and 40 units of E. coli RNase H (Takara Bio, Inc.) for 45 min at 30°C in a final volume of 25 µl. The effect of this treatment on RNase P activity in the extracts was determined by examining 5′-end processing of ^32^P-labeled E. coli precursor tRNA^Tyr^ and separation of the cleavage products in an 8% sequencing gel. An aliquot of 15 µl from each treated extract was then tested for in vitro transcription of mini-rRNA and small noncoding RNA genes as described above.
